# A Vascular-appearing Spindle Cell Xanthogranuloma in a Child

**DOI:** 10.7759/cureus.2595

**Published:** 2018-05-08

**Authors:** Daniel C Morse, Jaime A Tschen, Micheal R Migden, Sirunya Silapunt

**Affiliations:** 1 Mcgovern Medical School, University of Texas Mcgovern Medical School at Houston, Houston, USA; 2 Dermpath, St Joseph Dermpath, Houston, USA; 3 Departments of Dermatology and Head and Neck Surgery, The University of Texas Md Anderson Cancer Center, Houston, Texas; 4 Dermatology, University of Texas Mcgovern Medical School at Houston, Houston, USA

**Keywords:** xanthogranuloma, pediatrics

## Abstract

Spindle cell xanthogranuloma is a rare variant of juvenile xanthogranuloma that most commonly presents in adults as papulonodules. We describe a vascular-appearing case of spindle cell xanthogranuloma on the nose of a 10-year-old boy. The lesion was a dark red, well-demarcated, dome-shaped papule. Histopathology revealed spindle-shaped histiocytes in a storiform pattern that stained positive for cluster of differentiation 68 (CD68) and the nuclear antigen Ki-67. No vascular features were found. To our knowledge, this is the first reported spindle cell xanthogranuloma to mimic an angiomatous lesion.

## Introduction

Juvenile xanthogranuloma (JXG) is a benign proliferative disorder of dermal histiocytes that typically presents as yellowish to reddish papulonodules [1]. Spindle cell xanthogranuloma (SCXG) is a rare form of JXG and most often occurs in adulthood. Here, we present a pediatric and vascular-appearing case of SCXG.

## Case presentation

**Figure 1:**
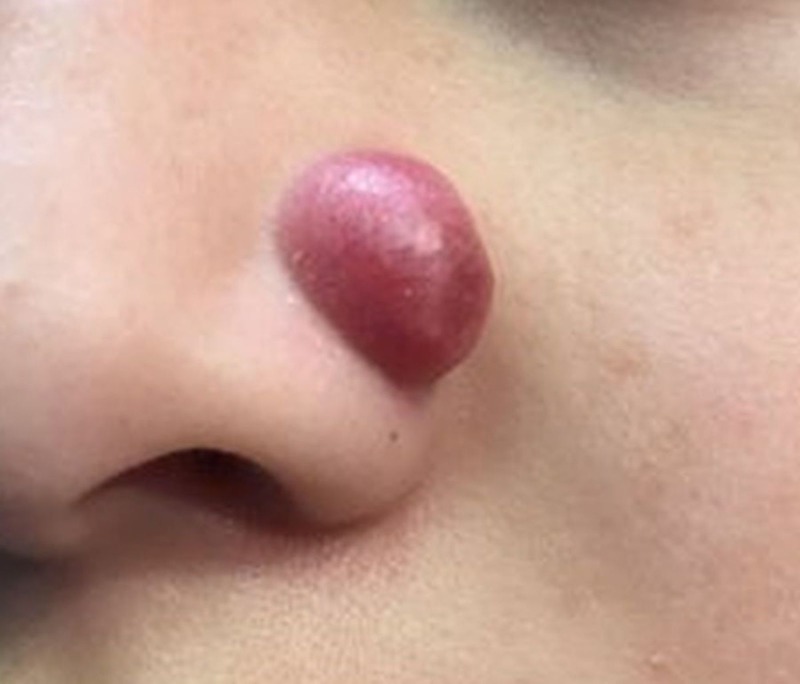
A 13-mm well-demarcated, dome-shaped, dark red nodule on the left ala

A 10-year-old male patient presented with a 13-mm well-demarcated, dome-shaped, dark red nodule on the left ala (Figure 1). It had been present for eight months. During that time, it had increased in size and bled. The lesion received no prior treatment. The remainder of the physical exam was unremarkable.

**Figure 2:**
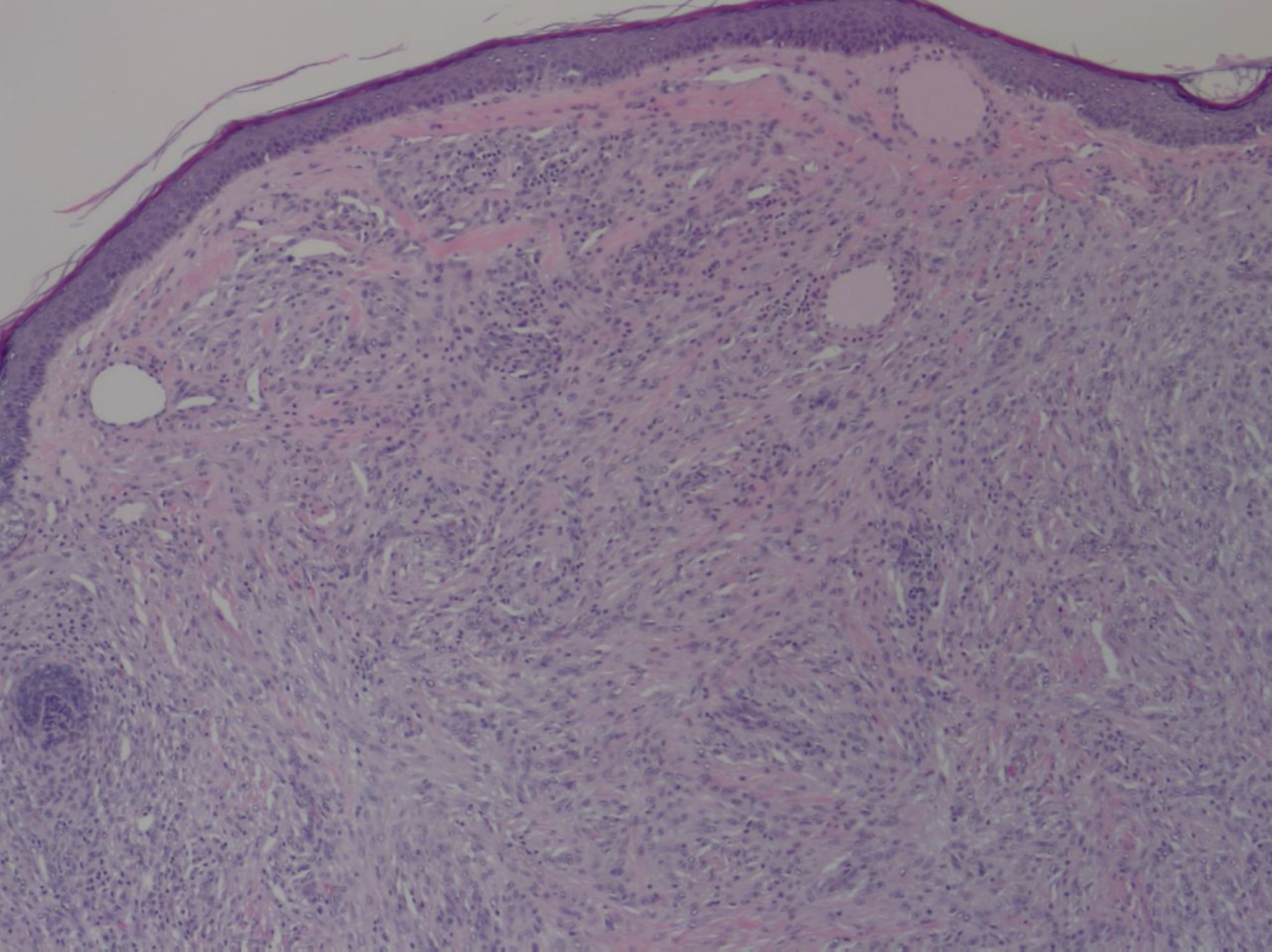
Diffuse proliferation of spindle cells in a storiform pattern Hematoxylin-eosin stain, original magnification 200x

**Figure 3:**
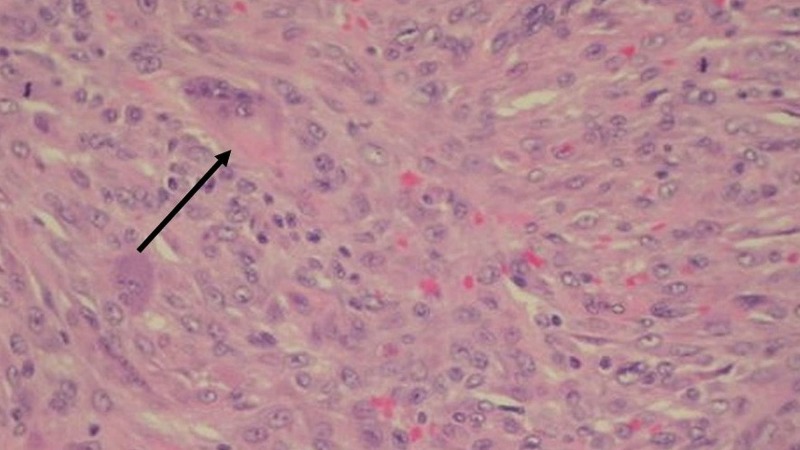
Dense proliferation of spindle-shaped histiocytes in the dermis, and a few multinucleated giant cells Arrow pointing to multinucleated giant cells. Hematoxylin-eosin stain, original magnification 400x

**Figure 4:**
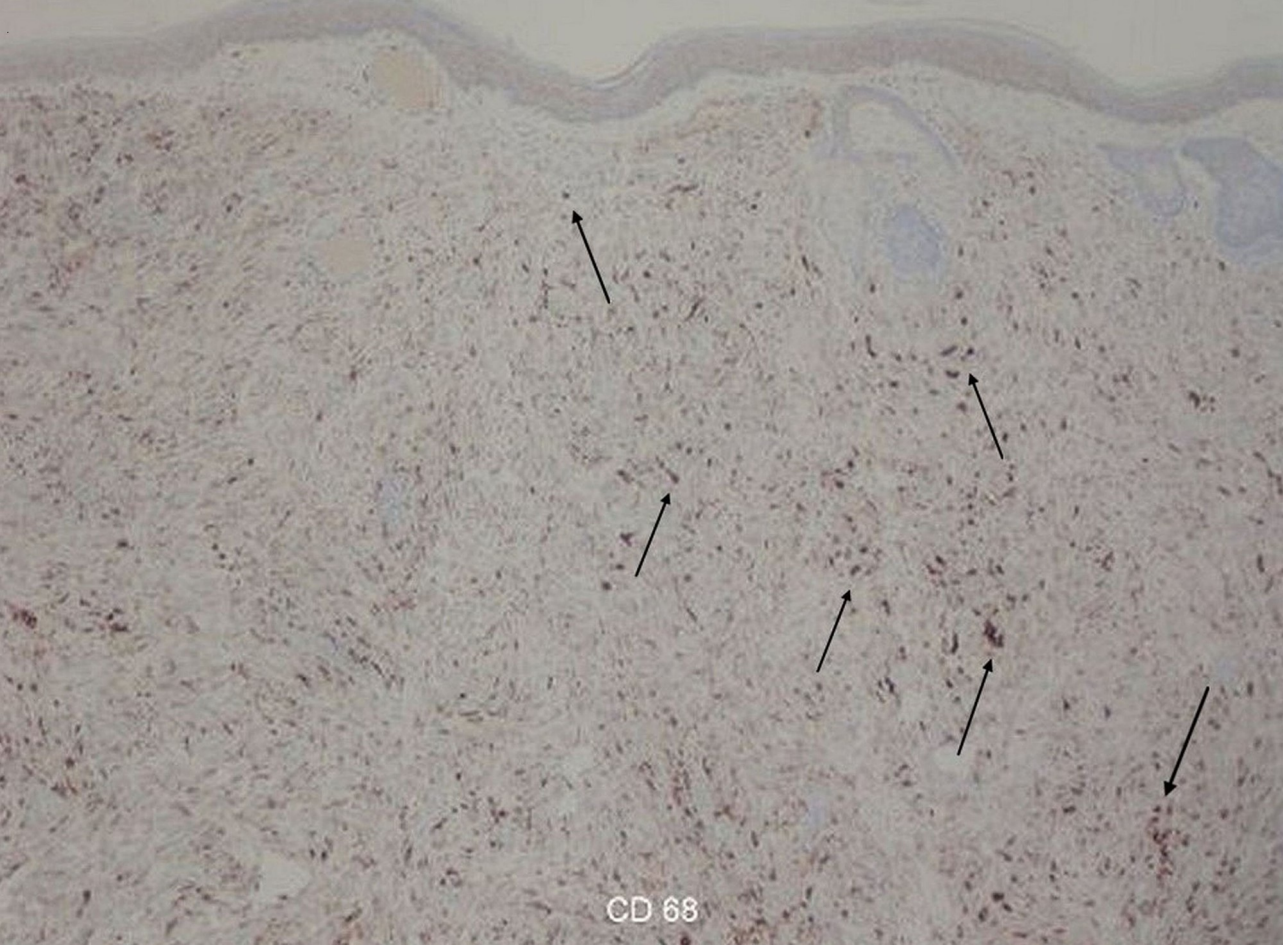
Diffuse infiltrate of spindle cells stained positive for CD68 (100x) Arrows showing positive cluster of differentiation 68 (CD68) staining.

A shave biopsy was performed, and histopathology revealed a diffuse infiltrate of spindle-shaped histiocytes in a storiform pattern (Figure 2), few multinucleated giant cells, scattered lymphocytes, and eosinophils (Figure 3). Immunohistochemical studies showed tumor cells positive for cluster of differentiation 68 (CD68) and the proliferation marker Ki-67 (Figure 4). The lesion was negative for S-100 protein, anti-melanoma antibody (HMB45), protein Melan-A, and smooth muscle actin (SMA). These histologic features supported the diagnosis of SCXG. The nodule resolved spontaneously several months later.

## Discussion

SCXG is a rare variant of JXG, originally described in 1995 by Zelger et al. who reported 12 solitary cases of SCXG [2]. Since 1995, only a few reports of SCXG have been described [3-5]. A literature review of previous case reports, including our report, is summarized in Table 1. SCXG classically presents as brownish to yellowish papulonodules involving the head, neck, upper trunk, and extremities in decreasing occurrence [2]. SCXG most often affects those between the ages of 20 - 40 years without preference for gender [2].

**Table 1:** Literature review of reported cases of SCXG SCXG: spindle cell xanthogranuloma; n/a: not available

Case No.	Author, year	Age (years)	Gender	Location	Size (mm)	Color	Recurrence
1	Zelger BW et al., 1995	11	F	Chin	n/a	n/a	No
2	Zelger BW et al., 1995	27	F	Neck	n/a	n/a	n/a
3	Zelger BW et al., 1995	21	M	Occiput	n/a	n/a	No
4	Zelger BW et al., 1995	59	M	Back	n/a	n/a	No
5	Zelger BW et al., 1995	18	F	Eyebrow	n/a	n/a	n/a
6	Zelger BW et al., 1995	31	F	Upper Trunk	n/a	n/a	No
7	Zelger BW et al., 1995	38	F	Abdomen	n/a	n/a	No
8	Zelger BW et al., 1995	41	M	Neck	n/a	n/a	No
9	Zelger BW et al., 1995	29	F	Back	n/a	n/a	No
10	Zelger BW et al., 1995	24	M	Calf	n/a	n/a	No
11	Zelger BW et al., 1995	54	F	Thigh	n/a	n/a	n/a
12	Zelger BW et al., 1995	15	M	Lower Arm	n/a	n/a	No
13	DeStafeno JJ et al., 2002	3	M	Eyelid	7x7	Yellowish Brown	n/a
14	Kim CR et al., 2012	0.92 (11 months)	F	Occiput	n/a	Yellowish Brown	n/a
15	Nakamura Y et al., 2013	10	F	Hip	10x5	Dark Red	No
16	Morse DC et al., 2018	10	M	Nose	13	Dark Red	No

In contrast to the typical SCXG presentation of brownish to yellowish papules appearing in adulthood, we describe a pediatric case of SCXG that presented with dark red vascular features appearing similar to a hemangioma. The histopathology failed to reveal vascular features and confirmed the diagnosis of SCXG. 

Spitz nevus (SN) was also high on our differential diagnosis since it also presents as a rapidly growing reddish nodule in children [6]. Nakamura et al. reported a case of SCXG in a 10-year-old, initially diagnosed as an SN due to the nodule’s dark red to bluish clinical appearance and peripheral blue background with white streaks evident upon dermoscopy [5]. However, histologic features of SN were not seen in our case. 

Histological examination of SCXG typically reveals Touton-type multinucleated giant cells and spindle-shaped histocytes in a storiform pattern [2]. The macrophage and dendritic cell ancestry of SCXG is confirmed through immunohistochemistry as SCXG stains positive for histiocyte markers: mature macrophage marker monoclonal antibody (HAM-56), CD68, and Factor XIIIa [2-3]. SCXG is histologically similar to progressive nodular histiocytosis; both tumors display a predominance of spindle cells in a storiform arrangement and multinucleated giant cells [2, 4]. However, progressive nodular histiocytosis appears in a disseminated pattern in the elderly, which is markedly different from the presentation of SCXG [2, 4]. Dermoscopy findings of SCXG appear as an orange-yellow structureless pattern with an erythematous border [7]. The tumors usually resolve spontaneously in six to 36 months [1].

## Conclusions

SCXG is a rare form of JXG that may clinically masquerade as various other neoplasms, including angiomatous lesions and Spitz nevi.
